# Impact of age and mean intracranial pressure on the morphology of intracranial pressure waveform and its association with mortality in traumatic brain injury

**DOI:** 10.1186/s13054-025-05295-w

**Published:** 2025-02-17

**Authors:** Magdalena Kasprowicz, Cyprian Mataczyński, Agnieszka Uryga, Adam I. Pelah, Eric Schmidt, Marek Czosnyka, Agnieszka Kazimierska, CENTER-TBI High-Resolution ICU (HR ICU) Sub-Study Participants and Investigators, Audny Anke, Ronny Beer, Bo-Michael Bellander, Erta Beqiri, Andras Buki, Manuel Cabeleira, Marco Carbonara, Arturo Chieregato, Giuseppe Citerio, Hans Clusmann, Endre Czeiter, Marek Czosnyka, Bart Depreitere, Ari Ercole, Shirin Frisvold, Raimund Helbok, Stefan Jankowski, Daniel Kondziella, Lars-Owe Koskinen, Ana Kowark, David K. Menon, Geert Meyfroidt, Kirsten Moeller, David Nelson, Anna Piippo-Karjalainen, Andreea Radoi, Arminas Ragauskas, Rahul Raj, Jonathan Rhodes, Saulius Rocka, Rolf Rossaint, Juan Sahuquillo, Oliver Sakowitz, Peter Smielewski, Nino Stocchetti, Nina Sundstrom, Riikka Takala, Tomas Tamosuitis, Olli Tenovuo, Andreas Unterberg, Peter Vajkoczy, Alessia Vargiolu, Rimantas Vilcinis, Stefan Wolf, Alexander Younsi, Frederick A. Zeiler

**Affiliations:** 1 Neuroengineering Lab, Department of Biomedical Engineering, Faculty of Fundamental Problems of Technology, Wroclaw University of Science and Technology, Wybrzeze Wyspianskiego 27, 50-370 Wroclaw, Poland; 2 Department of Computer Engineering, Faculty of Information and Communication Technology, Wroclaw University of Science and Technology, Wroclaw, Poland; 3 Division of Neurosurgery, Department of Clinical Neurosciences, Addenbrooke’s Hospital, University of Cambridge, Cambridge, UK; 4 Department of Neurosurgery, University Hospital of Toulouse, Toulouse, France; 5 Department of Physical Medicine and Rehabilitation, University Hospital Northern Norway, Tromsö, Norway; 6 Neurological Intensive Care Unit, Department of Neurology, Medical University of Innsbruck, Innsbruck, Austria; 7 Department of Neurosurgery and Anesthesia and Intensive Care Medicine, Karolinska University Hospital, Stockholm, Sweden; 8 NeuroIntensive Care, Niguarda Hospital, Milan, Italy; 9 Department of Neurosurgery, Medical School, University of Pecs, Hungary and Neurotrauma Research Group, Janos Szentagothai Research Centre, University of Pecs, Pecs, Hungary; 10 Brain Physics Lab, Division of Neurosurgery, Department of Clinical Neurosciences, University of Cambridge, Addenbrooke’s Hospital, Cambridge, UK; 11 Neuro ICU, Fondazione IRCCS Ca Granda Ospedale Maggiore Policlinico, Milan, Italy; 12 NeuroIntensive Care Unit, Department of Anesthesia and Intensive Care, ASST Di Monza, Monza, Italy; 13 School of Medicine and Surgery, Universita Milano Bicocca, Milan, Italy; 14 Department of Neurosurgery, Medical Faculty, RWTH Aachen University, Aachen, Germany; 15 Department of Neurosurgery, University of Pecs and MTA-PTE Clinical Neuroscience MR Research Group and Janos Szentagothai Research Centre, University of Pecs, Hungarian Brain Research Program (Grant No. KTIA 13 NAP-A-II/8), Pecs, Hungary; 16 Department of Neurosurgery, University Hospitals Leuven, Leuven, Belgium; 17 Division of Anaesthesia, University of Cambridge, Addenbrooke’s Hospital, Cambridge, UK; 18 Department of Anesthesiology and Intensive Care, University Hospital Northern Norway, Tromsö, Norway; 19 Neurointensive Care, Sheffield Teaching Hospitals NHS Foundation Trust, Sheffield, UK; 20 Departments of Neurology, Clinical Neurophysiology and Neuroanesthesiology, Region Hovedstaden Rigshospitalet, Copenhagen, Denmark; 21 Department of Clinical Neuroscience, Neurosurgery, Umea University, Umeå, Sweden; 22 Department of Anaesthesiology, University Hospital of Aachen, Aachen, Germany; 23 Intensive Care Medicine, University Hospitals Leuven, Leuven, Belgium; 24 Department Neuroanesthesiology, Region Hovedstaden Rigshospitalet, Copenhagen, Denmark; 25 Helsinki University Central Hospital, Helsinki, Finland; 26 Department of Neurosurgery, Vall d’Hebron University Hospital, Barcelona, Spain; 27 Department of Neurosurgery, Kaunas University of Technology and Vilnius University, Vilnius, Lithuania; 28 Department of Anaesthesia, Critical Care and Pain Medicine NHS Lothian and University of Edinburg, Edinburgh, UK; 29 Klinik Fur Neurochirurgie, Klinikum Ludwigsburg, Ludwigsburg, Germany; 30 Department of Neurosurgery, University Hospital Heidelberg, Heidelberg, Germany; 31 Department of Pathophysiology and Transplantation, Milan University, and Neuroscience ICU, Fondazione IRCCS Ca Granda Ospedale Maggiore Policlinico, Milan, Italy; 32 Department of Radiation Sciences, Biomedical Engineering, Umea University, Umeå, Sweden; 33 Intensive Care Medicine, and Pain Management, Perioperative Services, Turku University Central Hospital and University of Turku, Turku, Finland; 34 Neuro-Intensive Care Unit, Kaunas University of Health Sciences, Kaunas, Lithuania; 35 Rehabilitation and Brain Trauma, Turku University Central Hospital and University of Turku, Turku, Finland; 36 Neurologie, Neurochirurgie Und Psychiatrie, Charite–Universitatsmedizin Berlin, Berlin, Germany; 37 Department of Neurosurgery, Kaunas University of Health Sciences, Kaunas, Lithuania; 38 Department of Neurosurgery, Charite–Universitatsmedizin Berlin, corporate member of Freie Universitat Berlin, Humboldt-Universitat Zu Berlin, and Berlin Institute of Health, Berlin, Germany; 39 Section of Neurosurgery, Department of Surgery, Rady Faculty of Health Sciences, University of Manitoba, Winnipeg, MB Canada

**Keywords:** Cerebrospinal compliance, Traumatic brain injury, Intracranial pressure, Pulse waveform morphology, Aging

## Abstract

**Background:**

Morphological analysis of intracranial pressure (ICP) pulse waveforms provides indirect information on cerebrospinal compliance, which might be reduced by space-occupying lesions but also by intracranial hypertension and aging. This study investigates the impact of age and mean ICP on the shape and amplitude of ICP pulse waveform in traumatic brain injury (TBI). Additionally, it explores the association between morphological parameters and mortality after TBI.

**Methods:**

ICP recordings from 183 TBI patients (median age: 50 (30, 61) years) from the CENTER-TBI database were retrospectively analyzed. ICP morphology was assessed using the artificial intelligence-based pulse shape index (PSI) and peak-to-peak amplitude of ICP pulse waveform (AmpICP). The impact of mean ICP, age, and their interaction on PSI and AmpICP were estimated using factorial ANOVA. To account for influence of disturbance in the intracranial volume on AmpICP and PSI, a multiple regression analysis was performed using age, mean ICP, and the Rotterdam CT score as explanatory variables. The associations of AmpICP and PSI with six-month mortality were assessed using the area under the ROC curve (AUC).

**Results:**

Age had a predominant influence on PSI (*p* < 0.01), accounting for 33.1% of its variance, while mean ICP explained 6.6% (*p* < 0.01). Conversely, mean ICP primarily affected AmpICP (*p* < 0.01), explaining 22.8% of its variance, with age contributing 8.0% (*p* < 0.01). A combined effect of age and mean ICP on AmpICP (*p* = 0.01) explained 11.7% of its variance but did not influence PSI. After accounting for Rotterdam CT score, the results remained consistent, indicating that advanced age has the strongest impact on PSI (β = 0.342, *p* < 0.01) while elevated mean ICP has dominant influence on AmpICP (β = 0.522, *p* < 0.01). Both AmpICP and PSI were moderately associated with mortality (AUC: 0.76 and 0.71, respectively).

**Conclusions:**

AmpICP and PSI capture distinct aspects of cerebrospinal compliance. PSI appears to reflect age-related stiffening of the cerebrovascular system, while AmpICP, influenced by mean ICP, indicates acute volume compensatory changes. Combined, they provide a more comprehensive assessment of cerebrospinal volume–pressure compensation. Both morphological metrics are associated with mortality after TBI. As cerebrospinal compliance declines with age, older TBI patients become more susceptible to uncontrolled rises in ICP, which can worsen their outcome.

**Supplementary Information:**

The online version contains supplementary material available at 10.1186/s13054-025-05295-w.

## Background

Cerebrospinal compliance refers to the ability of the cerebrospinal system to accommodate rises in volume without significant increases in intracranial pressure (ICP) [1]. When the pressure–volume reserve approaches a critical level, defined as switching from the linear to the exponential shape of cerebrospinal pressure–volume curve [2], compliance drops sharply, and even small increases in volume can result in large increases in ICP. Age is an important factor that can modify this relationship [3–7]. With aging, the brain undergoes structural changes: brain tissue becomes more rigid [8], cerebrospinal fluid (CSF) absorption decreases [9], and cerebral vessels stiffen, leading to more pronounced pulsatile arterial pressure [10, 11]. These changes can reduce cerebrospinal compliance. Therefore, monitoring of cerebrospinal compliance in patients after traumatic brain injury (TBI) can be crucial, particularly in older individuals whose system may have already become less compliant and more susceptible to uncontrolled increases in ICP, elevating the risk of poor outcome [12, 13].

Historically, the intracranial volume–pressure relationship was investigated by introducing a known volume perturbation to the system while recording ICP [1, 14]. Although informative, this method is impractical for routine clinical use and poses safety concerns. To obtain a more clinically useful method of compliance assessment, several techniques based on analysis of the pressure response to naturally occurring cardiac-induced volume changes, reflected in the ICP pulse waveform, have been proposed. An inherent drawback of this methodology is the fact that cerebrospinal pulsatile volume load remains unknown, therefore compliance cannot be scaled in physical units (ml/mm Hg). Among the proposed indices, the ICP pulse amplitude (AmpICP) is the most widely used metric [15–17], with large amplitudes being associated with low compliance or increased cerebral arterial blood stroke volume [4, 5, 18, 19]. The relationship between AmpICP and mean ICP has been extensively studied, demonstrating its nonlinear nature [15, 20]. However, only a few studies have explored age-related changes in AmpICP [4, 5]. These studies suggest that AmpICP increases with age, and that this relationship is nonlinear.

Previous studies also suggest that the shape of the ICP pulse waveform may provide information on cerebrospinal compliance [21–25]. Decreasing compliance is associated with a progressive change in the pulse shape from a triphasic, saw-tooth pattern to a rounded or triangular wave with only one defined peak [26]. Recently, we introduced an artificial intelligence-based measure called the pulse shape index (PSI) [27–30] which is independent of AmpICP and pulse duration and allows for continuous tracking of changes in ICP pulse morphology. Our studies showed that PSI is significantly higher in TBI patients with poor outcomes [30, 31], correlates with volumetric imbalance represented by the presence of midline shift and mass lesions [28] and is useful for early prediction of life-threatening ICP crises [32, 33]. However, the relationship between PSI and age in TBI has not yet been determined.

Despite studies suggesting a link between the features of the ICP pulse waveform and cerebrospinal compliance [5, 22, 23], it remains unclear whether the amplitude and shape of the ICP pulse waveform convey corresponding information and could be used interchangeably, or if they complement each other and should be studied jointly. Therefore, in this study, we aim to conduct a comprehensive analysis of how age and mean ICP as well as their combination affect ICP pulse waveform-derived metrics, specifically AmpICP and PSI, in a large cohort of TBI patients. Additionally, we aim to examine the association of these morphological metrics with six-month mortality in TBI patients.

## Materials and methods

### Data acquisition

This study was conducted as a retrospective analysis of data collected in the high-resolution sub-study of the CENTER-TBI project (https://www.center-tbi.eu/; ClinicalTrials.gov identifier NCT02210221), with approval from the CENTER-TBI committee (Approval No. 359). The data were collected between 2015 and 2018 from 21 participating European centers involved in the CENTER-TBI project. All patients enrolled in the CENTER-TBI project were consistently treated according to the Brain Trauma Foundation guidelines, including recommendations for invasive ICP monitoring.

ICP was measured using intraparenchymal strain gauge probes (Codman ICP MicroSensor, Codman & Shurtleff Inc., Raynham, MA, USA) or parenchymal fiber optic pressure sensors (Camino ICP Monitor, Integra Life Sciences, Plainsboro, NJ, USA). The signal was recorded with sampling frequency of 100 Hz or higher using ICM + software (Cambridge Enterprise Ltd., Cambridge, UK) and/or Moberg CNS Monitor (Moberg Research Inc., Ambler, PA, USA). Data for the CENTER-TBI study were collected through Quesgen e-CRF (Quesgen Systems Inc., USA), hosted on the INCF platform and extracted via the INCF Neurobot tool (INCF, Sweden). Version CENTER Core 3.0 of the CENTER-TBI dataset was used in this study.

### Ethical approval/Informed consent

The CENTER-TBI study (European Commission grant 602150) was conducted in accordance with all relevant laws of the European Union if directly applicable or of direct effect and all relevant laws of the country where the recruiting sites were located, including but not limited to, the relevant privacy and data protection laws and regulations (the “Privacy Law”), the relevant laws and regulations on the use of human materials, and all relevant guidance relating to clinical studies from time to time in force including, but not limited to, the ICH Harmonised Tripartite Guideline for Good Clinical Practice (CPMP/ICH/135/95) (“ICH GCP”) and the World Medical Association Declaration of Helsinki entitled “Ethical Principles for Medical Research Involving Human Subjects.” Informed consent by the patients and/or the legal representative/next of kin was obtained, accordingly to the local legislations, for all patients recruited in the Core Dataset of CENTER-TBI and documented in the e-CRF. Ethical approval was obtained for each recruiting site from the appropriate local ethics committee, and the full list of approvals is available on the website: https://www.center-tbi.eu/project/ethical-approval.

### Study population

**Fig. 1:**
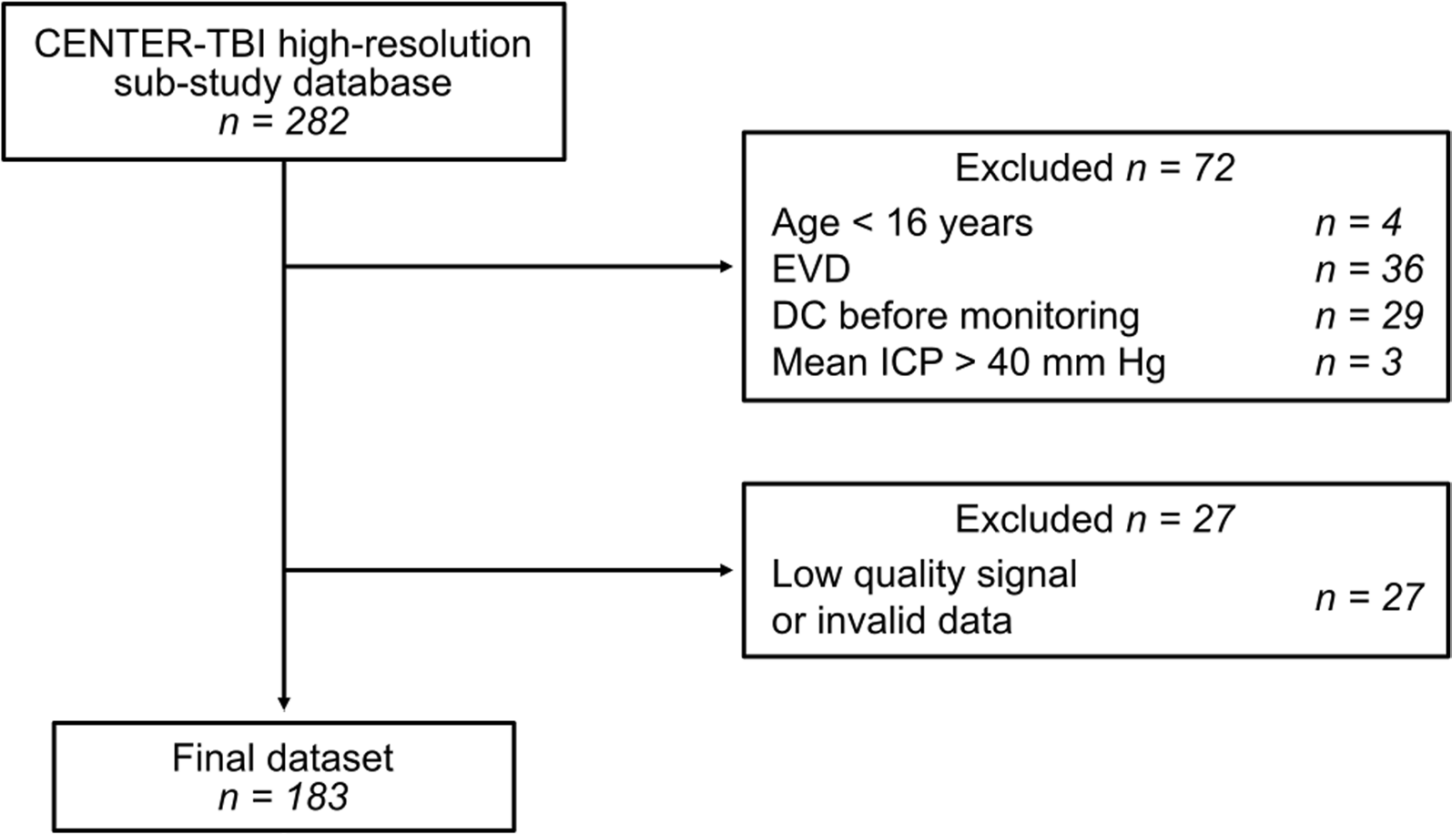
Selection criteria for the final patient dataset included in the study. EVD—external ventricular drain, DC—decompressive craniectomy, ICP—intracranial pressure, n—number of patients

The original dataset included 282 patients. The flowchart with selection criteria is presented in Fig. 1. Patients with ICP measured through external ventricular drains (EVDs) were excluded as the ICP pulse waveform could not be evaluated during CSF drainage periods. Additionally, patients who underwent decompressive craniectomy (DC) before ICP monitoring began were excluded due to alterations in the intracranial pressure–volume relationship caused by the removal of a portion of the skull bone. If DC took place during the monitoring period, the ICP signal was analyzed up to the moment of surgery. To avoid bias, patients in terminal condition with mean ICP > 40 mm Hg, reflecting extreme intracranial hypertension, were excluded as their inclusion could have influenced the statistical analysis, potentially skewing the results and limiting their applicability to the broader TBI population. A detailed summary of the study population is presented in the Results section.

### Computed tomography and outcome assessment

For each patient, the computed tomography (CT) scan performed directly prior to the start of monitoring was used for Rotterdam score assessment [34]. Follow-up status was assessed using the Glasgow Outcome Scale Extended (GOSE) score six months post-TBI. The patients were categorized based on their survival status into two groups: survivors or deceased, with the latter group identified based on in-hospital mortality records and GOSE score of 1 after six months.

### Morphological assessment of intracranial pressure pulse waveform

Every patient was characterized by the values of monitored parameters: mean ICP, AmpICP, and PSI averaged from the first seven days of recording.

AmpICP was calculated from the artifact-free parts of ICP recordings as the difference between the highest (maximum peak) and the lowest value (minimum valley) of the ICP pulse waveform within non-overlapping 2-s windows.

**Fig. 2:**
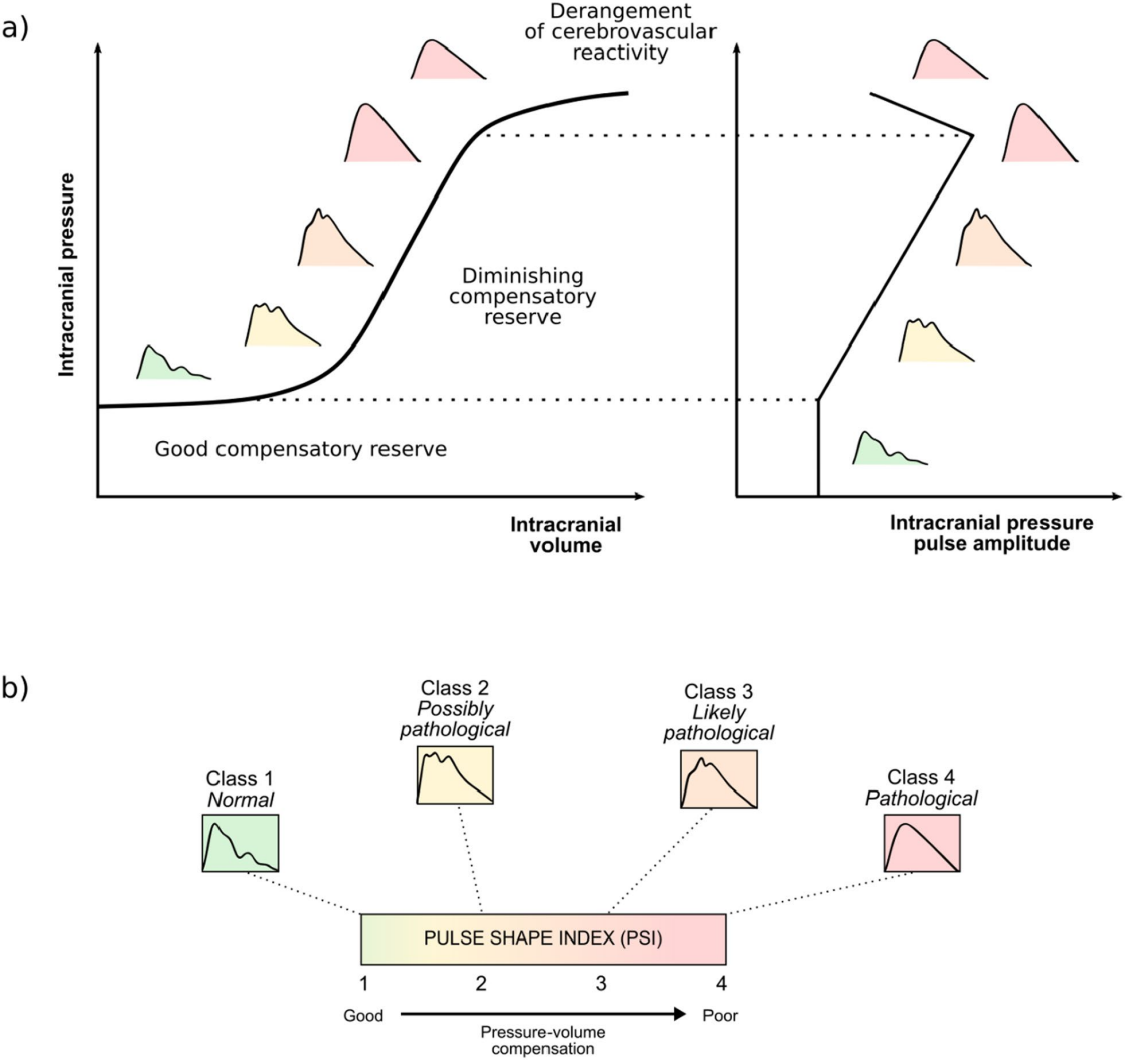
Morphological analysis of intracranial pressure (ICP). **a** Schematic representation of the relationship between intracranial volume, ICP, and changes in ICP pulse amplitude and shape. **b** Interpretation of the ICP pulse shape index (PSI)

PSI was calculated based on morphological classification of ICP pulses, conducted using a deep neural network model developed in our previous work [29]. This model identifies four types of pulse waveforms (see Fig. 2), *class 1*: normal waveform with dominant peak P1; *class 2*: potentially pathological waveform with increased prominence of peak P2, but P1 remaining higher than P3; *class 3*: likely pathological waveform with increased prominence of both P2 and P3; *class 4*: pathological rounded or triangular waveform with only one visible peak. Additionally, distorted waveforms or errors in pulse detection are marked by the model as artifacts to exclude invalid parts of the recording from further analysis. Prior to shape assessment, all pulses are normalized to a range of 0 to 1 to ensure they are independent of pulse amplitude and resampled to a uniform duration of 180 samples to eliminate the influence of heart rate variations.

Next, the classification results produced by the model (after removal of artefactual pulses) are used to calculate PSI in moving 5-min windows (window shift: 10 s) as the weighted sum of class numbers *i* and the fraction of pulses assigned to given class *p*_*i*_ according to the following formula:

1 $$PSI = \mathop \sum \limits_{i = 1}^{4} i \cdot p_{i}$$

As a result, PSI represents the average class number over a given period, enabling the capture of gradual changes in pulse shape on a continuous scale from 1 (indicating exclusively normal waveforms of class 1) to 4 (indicating exclusively pathologically altered waveforms of class 4); see Fig. 2.

### Statistical analysis

The normality of data distributions was tested using the Kolmogorov–Smirnov test. Factorial ANOVA was conducted to compare main effects of age (categorized into four levels: ≤ 30 years, (30–50] years, (50–61] years, and > 61 years) and mean ICP (categorized into four levels: ≤ 9 mm Hg, (9–12] mm Hg, (12–15] mm Hg, and > 15 mm Hg) as well as their interaction effects on either PSI or AmpICP. The threshold levels for categorization were selected based on the upwards-rounded values of the lower, median, and upper quartiles of the variables age and mean ICP, respectively, to ensure comparable number of patients in each group. The F-statistic was calculated for each main effect and for the interaction between the factors. The F-statistic is a ratio that compares the variance explained by the factor(s) or their interaction (systematic variance) to the variance due to random error (unsystematic variance). ANOVA results were presented as follows: F(df effect, df error) = F-statistic, p-value, where df denotes degrees of freedom. The effect size was assessed by partial η^2^. Post-hoc comparisons were performed using the Bonferroni test. Multiple regression analysis was performed to account for the influence of CT-based TBI classification on PSI or AmpICP with Rotterdam CT score, mean ICP, and age as explanatory (dummy) variables. The difference in the means of the analyzed parameters between patients who died and those who survived six months after TBI was assessed using the independent samples t-test. Logistic regression was applied to investigate the associations of AmpICP and PSI with six-month mortality. The model’s performance was evaluated using receiver operating characteristic (ROC) curves, with the area under the curve (AUC) serving as the evaluation metric. Data are presented as mean ± standard deviation (SD) unless indicated otherwise. The level of statistical significance was set at α = 0.05. Statistical analysis was performed using STATISTICA 13 (Tibco, Palo Alto, USA).

## Results

### Patient characteristics

Out of the full dataset of 282 patients, 183 were included in the analysis. 77% of the patients were men. The median age of the patients was 50 years, ranging from 16 to 85 years. Median Glasgow Coma Scale (GCS) sum score at admission was 7 and 66% of patients had GCS scores between 3 and 8, indicating severe head injury. The median GCS motor score was 4, reflecting moderate motor impairment, although the interquartile range (1–5) indicates variability in motor responses across patients. The median Rotterdam CT score was 3, and in 39% of patients, the Rotterdam CT score was higher than 3, suggesting moderate to severe intracranial injuries. CT data were unavailable for 34 patients. The ICU mortality rate was 9% (16 patients). Outcomes at six months were not available for 18 patients (10%). Among the 165 patients with known outcomes, 135 patients (82%) were classified as survivors, while 30 patients (18%) had died. Detailed clinical characteristics of the patients along with averaged values of parameters derived from multimodal monitoring are presented in Table 1.

**Table 1:** Summary characteristics of the patient cohort (total n = 183)

Clinical characteristics	
Parameter	Value
Age [years] *median [Q1, Q3]*	50 [30, 61]
Sex *n*	Male: 140, female: 43
GCS score *median [Q1, Q3]*	7 [4,10], NA: 10
GCS motor score *median [Q1, Q3]*	4 [1,5], NA: 4
Pupil reactivity *n*	Bilaterally nonreactive: 27, unilaterally nonreactive: 17, bilaterally reactive: 128, NA: 11
Rotterdam CT score* median [Q1, Q3]*	3 [3,4], NA: 34
ICU mortality *n*	Survived: 167, died: 16
Mortality after 6 months (GOSE score 1: deceased, 2–8: survived) *n*	Survived:135, died: 30, NA: 18
Parameters derived from multimodal monitoring (presented as *mean* ± *SD*)	
Parameter	Group-averaged value
Mean ICP [mm Hg]	12.2 ± 5.5
AmpICP [mm Hg]	8.0 ± 4.0
PSI [au]	2.5 ± 0.7

Data are presented as number of occurrences (n) and either as median [Q1, Q3] or as mean ± SD. Q1—first quartile, Q3—third quartile, SD—standard deviation, NA—data not available, GCS—Glasgow Coma Scale, ICU—intensive care unit, GOSE—Glasgow Outcome Scale Extended, ICP—intracranial pressure, AmpICP—peak-to-peak amplitude of ICP pulse waveform, PSI—pulse shape index, au—arbitrary units

### PSI vs mean ICP and age

**Fig. 3:**
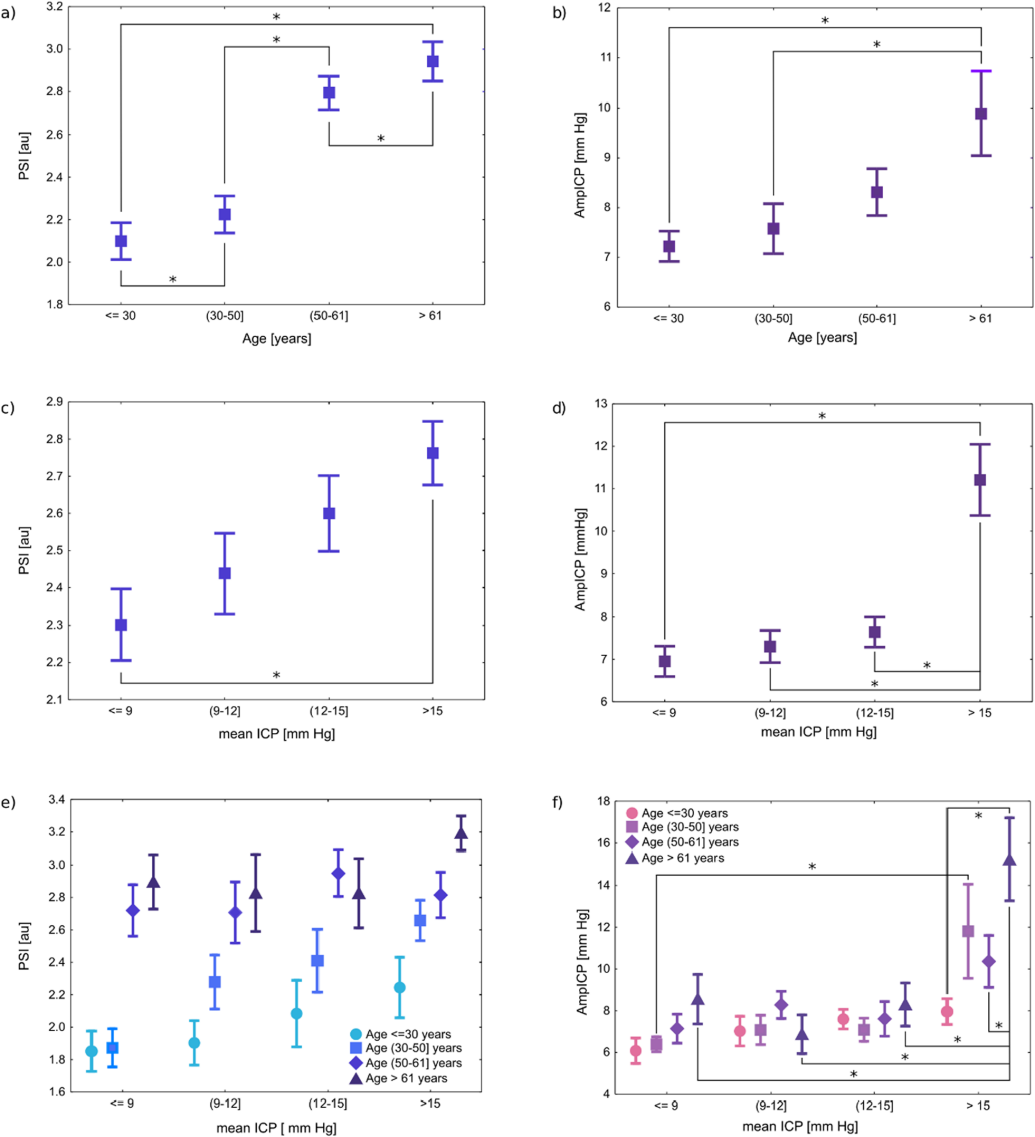
The impact of age and mean ICP on morphological metrics. The figure shows main effects of age (upper panel), mean ICP (middle panel), and the interaction effect of age and mean ICP (bottom panel) on PSI (left column) and AmpICP (right column). In subplot e, a consistent increasing trend between age and PSI is visible regardless of mean ICP level. In subplot f, a gradual trend of rising AmpICP with age is visible at mean ICP level above 15 mm Hg. Only statistically significant differences between age groups at a given level of ICP and between ICP levels within the same age group were annotated for clarity of subplot f. Since the interaction effect of mean ICP and age on PSI was insignificant (sublot e), post-hoc analyses were not performed in those cases. The central points in the graphs represent the means, and the vertical bars denote standard error. * denotes p_post-hoc_ < 0.01. ICP—intracranial pressure, AmpICP—peak-to-peak amplitude of ICP pulse waveform, PSI—pulse shape index, au—arbitrary units

Age and mean ICP effects on PSI were statistically significant (age: F(3, 167) = 25.107, *p* < 0.01, see Fig. 3a; mean ICP: F(3, 167) = 3.948, *p* < 0.01, see Fig. 3c). The main effects of age and mean ICP yielded effect sizes of 0.311 and 0.066, respectively, indicating that age explained 31.1% of the variance in PSI, while mean ICP explained only 6.6% of the variance. The interaction effect was not significant (F(9, 167) = 0.800, *p* = 0.6), indicating that there was no combined effect for age and mean ICP on PSI. PSI gradually increased with age independent of mean ICP level (Fig. 3e). After accounting for the influence of disturbance in the intracranial volume (assessed by Rotterdam CT score), the results remained consistent, indicating that advanced age has the strongest influence on PSI (β = 0.342, *p* < 0.01)—see Additional file 1.

### AmpICP vs mean ICP and age

The effect of mean ICP on AmpICP was statistically significant (F(3, 167) = 16.40, *p* < 0.01, see Fig. 3d). Age also had a significant impact on AmpICP (F(3, 167) = 4.84, *p* < 0.01, see Fig. 3b). The main effect of mean ICP had an effect size of 0.228, explaining 22.8% of the variance in AmpICP, while the main effect of age had an effect size of 0.080, accounting for 8.0% of the variance. The interaction between mean ICP and age was significant (F(9, 167) = 2.46, *p* = 0.01), with an effect size of 0.117, indicating that together they explained 11.7% of the variance in AmpICP (Fig. 3f).

Post-hoc analysis revealed that at mean ICP level above 15 mm Hg, AmpICP was significantly higher than at lower levels of mean ICP (Fig. 3d) and gradually increased with age (Fig. 3f). This gradual increase in AmpICP with age was not significant at lower levels of mean ICP. Additional correlation analysis performed for a subset of 44 patients with mean ICP higher than 15 mm Hg showed moderately strong association between AmpICP and age (r = 0.44, *p* < 0.01)— see Additional file 2. After accounting for the influence of disturbance in the intracranial volume (assessed by Rotterdam CT score), the results confirmed that elevated mean ICP has the strongest impact on AmpICP (β = 0.522, *p* < 0.01)—see Additional file 1.

The number of patients in each subgroup, according to the defined thresholds, along with mean values and standard deviations of age and ICP are provided in the Supplementary Tables 3.1–3.3 included in the Additional file 3.

### Associations of morphological indices with outcome

Outcome information was not available for 18 patients. Among the remaining 165 patients, 30 died six months after TBI. Patients who died had higher mean ICP than those who survived, but this difference was on the border of statistical significance (14.8 ± 7.8 vs 11.8 ± 4.9 [mm Hg], *p* = 0.05). Both AmpICP and PSI were also higher in patients who died (AmpICP: 11.7 ± 5.7 vs 7.6 ± 2.8 [mm Hg], *p* < 0.01; PSI: 2.9 ± 0.6 vs 2.4 ± 0.7 [au], *p* < 0.01). Patients who died were older that those who survived (63 ± 15 vs 45 ± 18 [years], *p* < 0.01).

Both AmpICP and PSI independently showed significant associations with mortality six months after TBI: AmpICP had a good association (χ^2^(1) = 24.45, *p* < 0.01, AUC = 0.76), as did PSI (χ^2^(1) = 12.60, *p* < 0.01, AUC = 0.71). Age and mean ICP were also linked to mortality, with age showing a good association (χ^2^(1) = 25.88, *p* < 0.01, AUC = 0.77) and mean ICP showing a moderate association (χ^2^(1) = 6.41, *p* = 0.01; AUC = 0.63). A multivariate logistic model that included all four parameters (AmpICP, PSI, mean ICP, and age) found that only AmpICP and age were significantly associated with mortality (χ^2^(2) = 39.7, *p* < 0.01), achieving an AUC of 0.83. PSI and mean ICP were found to be redundant in this model.

Similar associations between ICP pulse waveform morphology metrics and poor outcome, defined as GOSE ≤ 4 assessed six months post-injury, were also observed (Additional file 4).

## Discussion

In this study we assessed cerebrospinal volume–pressure compensatory reserve based on morphological analysis of ICP pulse waveform. This method relies on evaluation of the pressure response to volume changes occurring naturally during the cardiac cycle. As ICP is commonly measured during neurocritical care of TBI patients, this approach poses no additional risks to the patient. We found that age primarily influenced the shape of the ICP pulse waveform whereas mean ICP had a lesser effect on shape. On the other hand, AmpICP was primarily affected by mean ICP with minimal influence of age. However, when analyzing the combined effects of both age and mean ICP, the impact of age on AmpICP became more pronounced when ICP was elevated.

The morphology of the ICP pulse waveform is governed by pulsatile cerebral arterial inflow, cerebral venous outflow, and the mechanoelastic properties of the cerebrospinal space [35, 36]. With aging, significant structural and functional remodelling occurs within the cerebrovascular system, as brain tissue loses compressibility [8] and CSF circulation decreases [4, 9, 37]. Additionally, cerebral arteries become stiffer in older individuals, resulting in more pronounced pulsatile pressure [11]. These changes may collectively alter the morphology of ICP pulses and lead to an increase in PSI with age. While PSI increased further with rising ICP, the impact of rising ICP on waveform morphology was weak. Patients who died had significantly higher PSI values, and altered shapes of ICP pulse waveforms were observed even at low ICP levels (see Additional file 4). Those patients were also older than those who survived.

A previous study showed no effect of age on AmpICP in young patients up to about 35 years old but a significant increase in AmpICP in older patients, particularly in those over 60 years old [5]. We found that the effect of age on AmpICP became more evident when ICP was elevated (above 15 mm Hg). This suggests that age should be considered when interpreting the amplitude–pressure relationship. In patients who did not survive, the increase in AmpICP with mean ICP was steeper, indicating that rising ICP results in a more pronounced increase in amplitude compared to patients who survived (see Additional file 4). Additionally, AmpICP was elevated in patients who died, regardless of age.

Our findings suggest that AmpICP and PSI capture different aspects of cerebrospinal pressure–volume compensation. PSI is sensitive to age-related stiffening of the cerebrospinal system and can reflect gradual decline in compliance associated with aging. AmpICP, being mostly dependent on mean ICP, can indicate more acute changes in the cerebrospinal system's ability to compensate for volume changes. Together, these two metrics may offer a more comprehensive assessment of cerebrospinal compliance. Both PSI and AmpICP show a significant association with six-month mortality following TBI. However, PSI, which incorporates age-related information, became redundant in a multivariate logistic model that already adjusts for age. Nevertheless, monitoring of both morphological parameters during the patients’ stay in the ICU can be clinically valuable, as it enables continuous, real-time tracking of dynamic changes in compliance. This approach potentially enables early identification of patients at risk of uncontrolled ICP elevation, allowing for preventive interventions to be implemented before substantial clinical deterioration occurs.

Earlier studies have suggested that cerebrospinal compliance may be reduced in elderly individuals [4, 5], potentially contributing to poorer outcomes observed in older patients following TBI. These studies primarily focused on indices describing AmpICP and their relationship with age, neglecting the influence of mean ICP levels. Only one study, which involved a limited sample of 30 TBI patients, conducted a combined analysis of mean ICP and age on cerebrospinal compliance [6]. However, in that study compliance assessment was performed using the Spiegelberg brain compliance monitor in which volumetric changes were induced via a periodically expanding intraventricular balloon. Although the Spiegelberg monitor has been used in clinical studies [38, 39], considerable technical issues with this methodology have been also reported [6, 40]. In contrast, in this study we focused solely on the analysis of high-resolution ICP recordings collected during ICU management of TBI patients.

### Limitations

The morphological classification scale is an approximation that does not encompass all possible ICP pulse shapes but allows for comparison of the overall shape observed in different patients; on the other hand, AmpICP is highly variable, and reference values are not available for TBI. We analyzed metrics averaged over the first seven days of monitoring for each patient. While this approach helps identify general associations, it does not account for critical events, such as prolonged intracranial hypertension. Incorporating additional analysis of ICP rises, for example through ICP dose calculations, could provide a more detailed understanding of the effects of AmpICP and PSI on patient outcomes. Moreover, we did not analyze the influence of either systemic arterial blood pressure (ABP) pulse waveform shape or anesthetic drugs, which can affect the amplitude and shape of ICP pulse waveforms and ultimately impact compliance assessment (as indicated by additional analyses of the effects of mean ABP and its pulse amplitude on ICP pulse metrics; see Additional file 5). One limitation of the statistical analysis is the inherent dependence of ICP waveform morphology on absolute ICP levels. While stratifying data by ICP ranges minimizes this confounding effect, some residual dependence may persist. However, we did not find multicollinearity between mean ICP, AmpICP, and PSI in the regression analysis, which suggests that the relationships between variables can be reliably interpreted. Next, we used thresholds tailored to our dataset instead of literature-based thresholds for age and mean ICP. While applying thresholds established in the literature might have increased the clinical applicability of our findings, this approach was not feasible due to the small sample sizes in specific subgroups. Tailoring the thresholds to our dataset was necessary to ensure a statistically robust analysis and draw conclusions that are both valid and representative of our study population. An additional limitation of our study is the imbalanced nature of the dataset, particularly in the analysis of mortality (18% mortality rate). Our primary objective was to explore associations between morphological metrics and clinical outcomes. Thus, we focused on the AUC as the primary performance metric to provide a robust and interpretable assessment of overall model performance. The values of additional metrics, such as sensitivity and specificity, can be found in Additional file 6.

Other ICP pulse-related indices, such as compensatory reserve index (RAP) [15], high frequency centroid [24], and higher harmonics centroid [25], were not studied along AmpICP and PSI as this would overload this study and make it far less comprehensible. Future research should aim to also include indices related to cerebrovascular reactivity for a detailed assessment of the relationship between aging-related changes in ICP pulse waveform and the cerebrovascular system.

## Conclusions

We found that the shape of the ICP pulse waveform is primarily influenced by age, whereas pulse amplitude depends largely on mean ICP, suggesting that AmpICP and PSI capture different aspects of cerebrospinal compliance. PSI reflects age-related stiffening of the cerebrovascular system, while AmpICP, driven by mean ICP, highlights acute changes in volume compensation. Both metrics are associated with six-month mortality after TBI. The combined monitoring of AmpICP and PSI offers insights into the timing and progression of changes in the intracranial pressure–volume compensatory reserve, facilitating early identification of patients at risk of uncontrolled ICP elevation. This may allow for preventive interventions before significant clinical deterioration occurs and emphasizes proactive management over reactive threshold-based treatment for TBI patients.

## Supplementary Information

Additional file1 (DOCX 187 KB)

Additional file2 (DOCX 17 KB)

Additional file3 (DOCX 218 KB)

Additional file4 (DOCX 31 KB)

Additional file5 (DOCX 17 KB)

Additional file6 (SVG 17 KB)

## Data Availability

The data that support the findings of this study belong to the CENTER-TBI project (https://www.center-tbi.eu/) but restrictions apply to the availability of these data, which were used under license for the current study (Approval No 359), and so are not publicly available. Access to the data can be obtained upon approval from the CENTER-TBI project committee. Source codes for the ICP pulse waveform classification plugin are available online: https://github.com/CMataczynski/ICMPWaveformClassificationPlugin.

## Abbreviations

AmpICP: Pulse amplitude of intracranial pressure
AUC: Area under the curve
CSF: Cerebrospinal fluid
CT: Computed tomography
DC: Decompressive craniectomy
EVD: External ventricular drain
GCS: Glasgow Coma Scale
GOSE: Glasgow Outcome Scale Extended
ICP: Intracranial pressure
ICU: Intensive care unit
PSI: Pulse shape index
ROC: Receiver operating characteristic (curve)
SD: Standard deviation
TBI: Traumatic brain injury
