# Supplementary material for: Impact of age and mean intracranial pressure on the morphology of intracranial pressure waveform and its association with mortality in traumatic brain injury
Source: Crit Care. 2025 Feb 17;29:78. doi: 10.1186/s13054-025-05295-w (PMC11834513; doi:10.1186/s13054-025-05295-w)
Supplement: Supplementary file 1 — Additional file1 (DOCX 187 KB) [file 13054_2025_5295_MOESM1_ESM.docx]

**Supplementary material 1**

*Multiple regression analysis*

A multiple regression analysis was conducted to examine whether age, mean intracranial pressure (mICP), and the Rotterdam CT score account for variations in PSI and AmpICP. Since mICP and age were categorized into four levels within the factorial ANOVA framework, and the Rotterdam CT score is a categorical variable, dummy variables were created with the reference categories defined as mICP ≤ 9 mm Hg, age ≤ 30 years, and Rotterdam score = 1, respectively. To detect multicollinearity, the variance inflation factor (VIF) was employed. Due to missing data on the Rotterdam score for 34 patients, the analysis was based on data from 149 TBI patients.

*PSI model*

The first regression model examined the collective effect of age, mean ICP, and Rotterdam CT score on PSI. The model accounted for approximately 35% of the variability in PSI (adjusted R²=0.351), which was statistically significant (F(11,137)=8.28, p<0.01), and indicated a large effect size (f²=0.541).

Individual contribution of explanatory variables:

- Age (reference: ≤ 30 years):

(30–50] years: β=-0.225, t=-2.525, p=0.01, VIF=1.8

(50–61] years: β=0.294, t=3.392, p<0.01, VIF=1.7

> 61 years: β=0.342, t=3.838, p<0.01, VIF=1.8

- Mean ICP (reference: ≤ 9 mm Hg):

>15 mm Hg: β=0.211, t=2.470, p=0.02, VIF=1.7

The intermediate levels of mean ICP (9–12 mm Hg, 12–15 mm Hg) did not significantly affect PSI.

- Rotterdam CT score (reference: 1):

Scores 2–6 did not significantly affect PSI.

The analysis showed that advanced age (above 61 years) had the strongest influence on PSI as reflected by the largest standardized beta coefficient (β=0.342). Elevated mean ICP (>15 mm Hg) also had a significant positive association with PSI. Neither the intermediate levels of mean ICP nor the Rotterdam CT scores had a significant effect on PSI. VIF values were all below 2, indicating no substantial multicollinearity concerns.

*AmpICP model*

The second regression model assessed the relationship between AmpICP and the same dummy predictors (age, mean ICP, and Rotterdam CT score). This model explained 20.6% of the variability in AmpICP (adjusted R²=0.206), which was statistically significant (F(11,137)=4.49, p<0.01), and indicated a medium effect size (f²=0.259).

Individual contribution of explanatory variables:

- Mean ICP (reference: ≤ 9 mm Hg):

> 15 mm Hg: β=0.522, t=5.52, p<0.01, VIF=1.7

- Age (reference: ≤ 30 years):

> 61 years: β=0.291, t=2.96, p<0.01, VIF=1.8

- Rotterdam CT score: (reference: 1):

Scores 2–6 did not significantly affect AmpICP.

In this model, mean ICP above 15 mm Hg demonstrated the strongest influence on AmpICP (β=0.522). While advanced age (above 61 years) also contributed significantly, the Rotterdam score did not affect AmpICP.

*Short discussion*

After accounting for the Rotterdam CT score, the main results remained consistent with those obtained from factorial ANOVA, indicating that advanced age has the strongest impact on PSI (age > 61 years: β=0.342, p<0.01), while elevated ICP exerts a dominant influence on AmpICP (mean ICP > 15 mm Hg: β=0.522, p<0.01). In fact, the Rotterdam CT score did not significantly influence either PSI or AmpICP. However, it should be noted that the Rotterdam CT score was assessed from the first CT scan, and the lack of volumetric imaging during ICP monitoring, combined with 18% missing data on the Rotterdam scale, represents a limitation of this analysis. We found that older patients had higher Rotterdam CT scores (see Supplementary Figure 1.1), but the values of the VIF factor were below 2, indicating no substantial multicollinearity concerns.


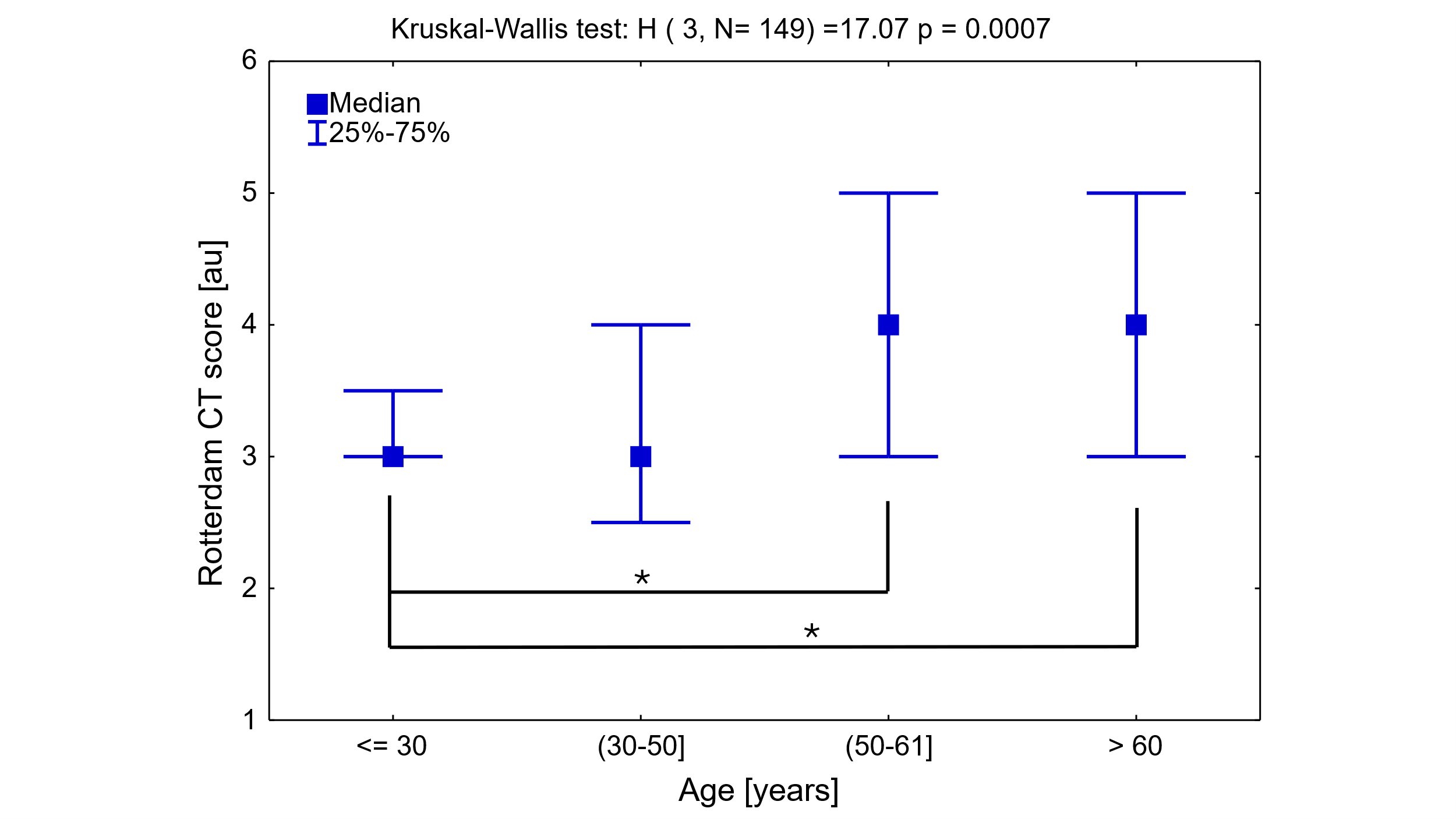


Supplementary Fig. 1.1. Differences in the Rotterdam CT score between the age-group categories (age: ≤ 30 years, n=44, age: (30–50] years, n=36, age: (51–61] years, n=39, age: > 60 years, n=30) * denotes p_post-hoc_ <0.01, n—number of cases in each age group, au—arbitrary units.
