# Supplementary material for: Impact of age and mean intracranial pressure on the morphology of intracranial pressure waveform and its association with mortality in traumatic brain injury
Source: Crit Care. 2025 Feb 17;29:78. doi: 10.1186/s13054-025-05295-w (PMC11834513; doi:10.1186/s13054-025-05295-w)
Supplement: Supplementary file 2 — Additional file2 (DOCX 17 KB) [file 13054_2025_5295_MOESM2_ESM.docx]

**Supplementary material 2**

*Correlations of PSI and AmpICP with age and mean ICP*

Correlation analysis performed using the Pearson correlation coefficient (r) showed that PSI was moderately strong correlated with age (Supplementary Fig. 2.1a, r=0.51, p<0.01), while AmpICP was weakly correlated with age (Supplementary Fig. 2.1b, r=0.27, p<0.01). Additional analysis performed for a subset of 44 patients with mean ICP higher than 15 mm Hg (the value of mean ICP above which an increase in AmpICP with age was observed; see Fig. 3f in main text of the article) showed moderately strong association between AmpICP and age (r=0.44, p<0.01). No relationship was found between age and mean ICP.

Both PSI and AmpICP were associated with mean ICP, with AmpICP showing a stronger positive correlation (Supplementary Fig. 2.1d, r=0.57, p<0.01) compared to PSI (Supplementary Fig. 2.1c, r=0.24, p<0.01). PSI and AmpICP were weakly correlated (r=0.34, p<0.01).


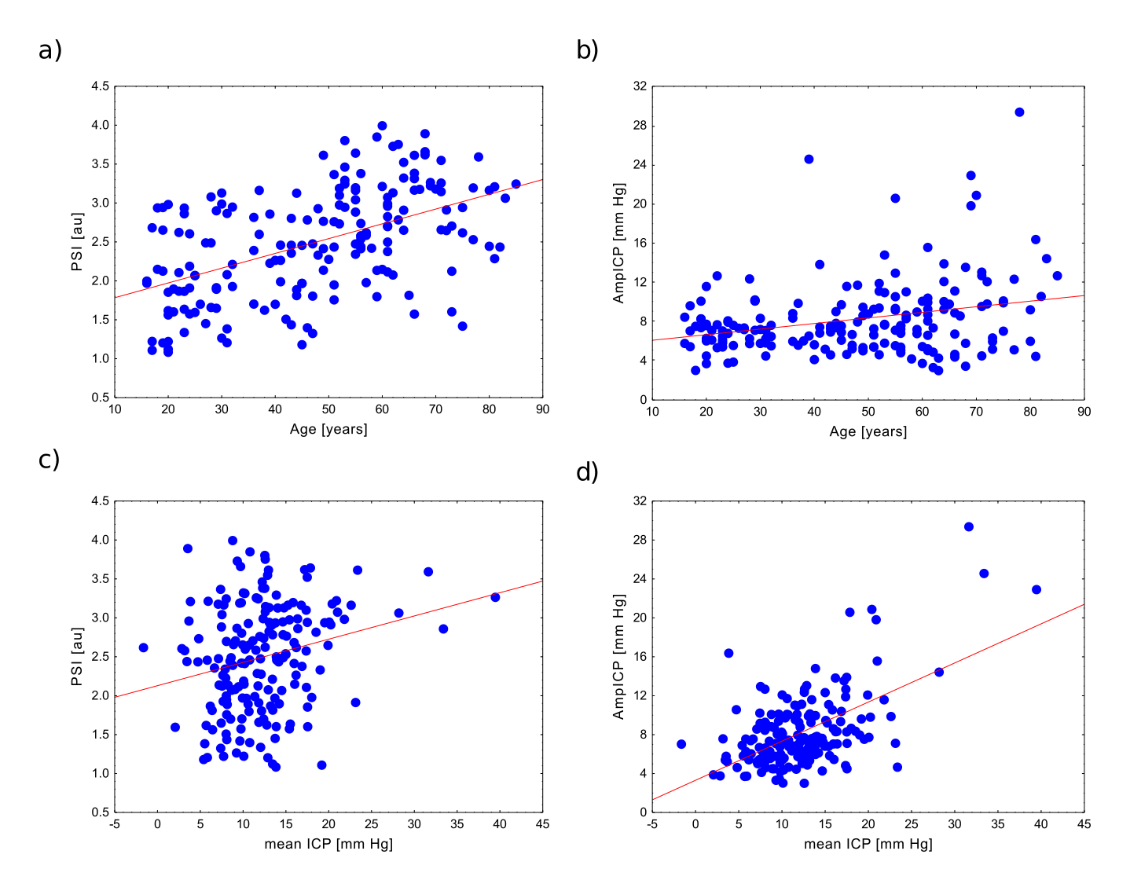


Supplementary Fig. 2.1 The relationships between pulse shape index (PSI; left panel) and peak-to-peak amplitude of intracranial pressure (ICP) pulse waveform (AmpICP; right panel) and age (subplots a and b) and mean ICP (subplots c and d). Blue dots indicate individual data points while the red lines indicate linear regression lines. au—arbitrary units

*Short discussion*

In line with previous studies [1–3], we observed an increase in AmpICP with mean ICP. A previous study showed no effect of age on AmpICP in young patients up to about 35 years old and a significant increase in AmpICP in older patients, with the most pronounced rise in those over 60 years old [4]. In our study, we observed a moderate but significant correlation between AmpICP and age; however, we did not find a stronger link between amplitude and age in older individuals. Instead, we discovered that the correlation with age becomes more significant when ICP is elevated (above 15 mm Hg).

In our study mean ICP was not correlated with age. However, a decrease in mean ICP with aging has been previously reported [5,6].

*References*

1. Czosnyka M, Guazzo E, Whitehouse M, Smielewski P, Czosnyka Z, Kirkpatrick P, et al. Significance of intracranial pressure waveform analysis after head injury. Acta Neurochir (Wien). 1996;138:531–42. doi: 10.1007/BF01411173

2. Avezaat CJJ, Van Eijndhoven JH, Wyper DJ. Cerebrospinal fluid pulse pressure and intracranial volume-pressure relationships. J Neurol Neurosurg Psychiatry. 1979;42:687–700. doi: 10.1136/jnnp.42.8.687

3. Szewczykowski J, Sliwka S, Kunicki A, Dytko P, Korsak-Sliwka J. A fast method of estimating the elastance of the intracranial system. J Neurosurg. 1977;47:19–26. doi: 10.3171/jns.1977.47.1.0019

4. Howells T, Lewén A, Sköld MK, Ronne-Engström E, Enblad P. An evaluation of three measures of intracranial compliance in traumatic brain injury patients. Intensive Care Med. 2012;38:1061–8. doi: 10.1007/s00134-012-2571-7

5. Czosnyka M, Balestreri M, Steiner L, Smielewski P, Hutchinson PJ, Matta B, et al. Age, intracranial pressure, autoregulation, and outcome after brain trauma. J Neurosurg. 2005;102:450–4. doi: 10.3171/jns.2005.102.3.0450

6. Pedersen SH, Lilja-Cyron A, Andresen M, Juhler M. The relationship between intracranial pressure and age—chasing age-related reference values. World Neurosurg. 2018;110:e119–23. doi: 10.1016/j.wneu.2017.10.086
