# Supplementary material for: Impact of age and mean intracranial pressure on the morphology of intracranial pressure waveform and its association with mortality in traumatic brain injury
Source: Crit Care. 2025 Feb 17;29:78. doi: 10.1186/s13054-025-05295-w (PMC11834513; doi:10.1186/s13054-025-05295-w)
Supplement: Supplementary file 3 — Additional file3 (DOCX 218 KB) [file 13054_2025_5295_MOESM3_ESM.docx]

**Supplementary material 3**

Tables present the number of patients and the mean ± standard deviation of age and intracranial pressure (ICP) in each subgroup, as determined by the specified threshold levels.

Supplementary Table 3.1. Distribution of patients’ numbers and mean age for each age range

| Age range [years] | n | Mean age ± SD [years] |
| --- | --- | --- |
| ≤ 30 | 48 | 22.9 ± 4.2 |
| (30–50] | 44 | 41.4 ± 5.8 |
| (50–61] | 47 | 56.2 ± 3.4 |
| > 61 | 44 | 70.9 ± 6.4 |

n–number of patients, SD–standard deviation

Supplementary Table 3.2. Distribution of patients’ numbers and mean intracranial pressure (ICP) for each ICP range

| ICP range [mm Hg] | n | Mean ICP ± SD [mm Hg] |
| --- | --- | --- |
| ≤ 9 | 51 | 6.6 ± 2.1 |
| (9–12] | 41 | 10.4 ± 0.8 |
| (12–15] | 47 | 13.3± 0.8 |
| > 15 | 44 | 19.4 ± 5.1 |

n–number of patients, SD–standard deviation

Supplementary Table 3.3. Distribution of patients’ numbers mean age and mean intracranial pressure (ICP) for each combination of age and ICP ranges

| ICP range  [mm Hg] | Age range  [years] | n | Mean age ± SD  [years] | Mean ICP ± SD  [mm Hg] |
| --- | --- | --- | --- | --- |
| ≤ 9 | ≤ 30 | 11 | 23.3 ± 3.9 | 6.6 ± 2.3 |
|  | (30–50] | 16 | 41.1 ± 5.8 | 7.1 ± 1.0 |
|  | (50–61] | 14 | 55.9 ± 3.8 | 6.6 ± 2.0 |
|  | > 61 | 10 | 74.8 ± 6.9 | 5.6 ± 3.3 |
| (9–12] | ≤ 30 | 10 | 22.4 ± 5.3 | 10.5 ± 0.9 |
|  | (30–50] | 10 | 39.9 ± 6.0 | 10.2 ± 0.7 |
|  | (50–61] | 10 | 56.7 ± 3.6 | 10.4 ± 1.0 |
|  | > 61 | 11 | 69.7 ± 6.1 | 10.3 ± 0.8 |
| (12–15] | ≤ 30 | 14 | 24.0 ± 3.6 | 13.5 ± 0.8 |
|  | (30–50] | 11 | 42.4 ± 6.2 | 13.2 ± 0.8 |
|  | (50–61] | 11 | 56.0 ± 3.6 | 13.1 ± 1.0 |
|  | > 61 | 11 | 66.8 ± 4.8 | 13.1 ± 0.7 |
| > 15 | ≤ 30 | 13 | 21.8 ± 4.1 | 17.9 ± 2.4 |
|  | (30–50] | 7 | 42.7 ± 5.8 | 20.1 ± 6.4 |
|  | (50–61] | 12 | 56.2 ± 3.2 | 17.4 ± 1.7 |
|  | > 61 | 12 | 72.3 ± 5.9 | 22.4 ± 7.3 |

n–number of patients, SD–standard deviation
