# Supplementary material for: Impact of age and mean intracranial pressure on the morphology of intracranial pressure waveform and its association with mortality in traumatic brain injury
Source: Crit Care. 2025 Feb 17;29:78. doi: 10.1186/s13054-025-05295-w (PMC11834513; doi:10.1186/s13054-025-05295-w)
Supplement: Supplementary file 4 — Additional file4 (DOCX 31 KB) [file 13054_2025_5295_MOESM4_ESM.docx]

**Supplementary material 4**

*Associations of morphological indices with mortality six months after TBI*

The results of the logistic regression model are presented in the main text of the article. Here, we present the differences between patients who died and those who survived in PSI and AmpICP with respect to mean ICP and age. In patients who survived, PSI gradually increased with mean ICP. However, in patients who died, PSI was already elevated at lower levels of mean ICP and did not show an increasing trend with rising ICP (Supplementary Fig. 4.1a). PSI gradually increased with advanced age for both survivors and those who died (Supplementary Fig. 4.1.b). In contrast to PSI, AmpICP demonstrated an increasing trend with rising mean ICP for both groups of patients: those who died and those who survived. However, the rise in AmpICP was steeper at higher levels of mean ICP for patients who died (Supplementary Fig. 4.1 c). There was no clear relationship between age and AmpICP for either the patients who died or those who survived (Supplementary Fig. 4.1 d).


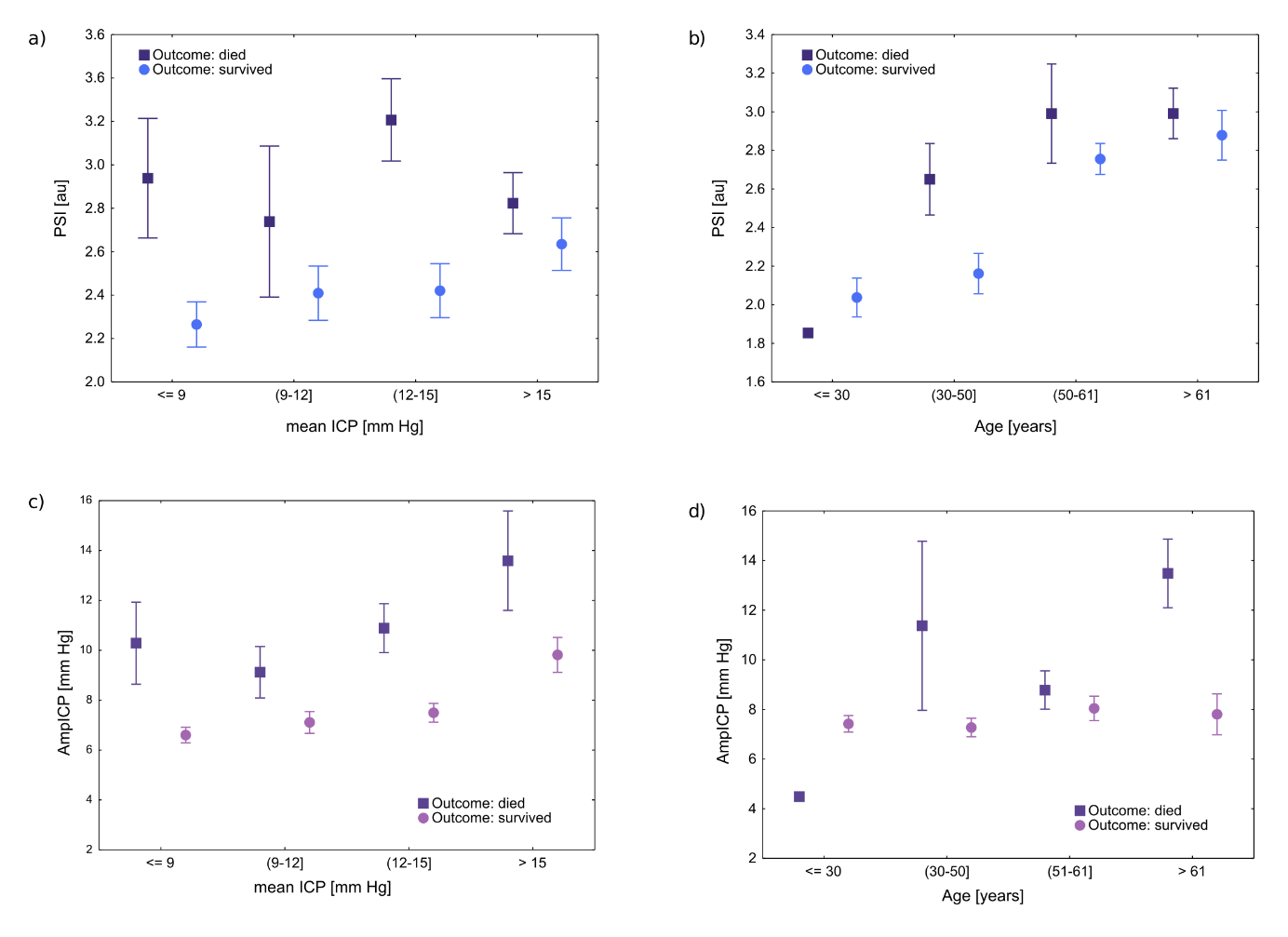


Supplementary Fig. 4.1 Differences in pulse shape index (PSI; upper panel) and peak-to-peak amplitude of intracranial pressure (ICP) pulse waveform (AmpICP; lower panel) accounting for mean ICP (subplots a and c) and age (subplots b and d) between patients who died and those who survived six months post-injury. The central points in the graph represent the means, and the vertical bars denote the standard error. au—arbitrary units

*Associations of morphological indices with poor outcome six months after TBI*

A poor functional outcome, defined as GOSE ≤ 4, was assessed at six months post-injury. Outcome information was not available for 18 patients. Among the remaining 165 patients, 88 had a poor outcome. Both PSI and AmpICP were higher in patients with poor outcome compared to those with good outcome (PSI: 2.6 ± 0.7 vs 2.4 ± 0.7 [au], p<0.02; AmpICP: 9.2 ± 4.3 vs 7.4 ± 3.0 [mm Hg], p<0.01). There were no significant differences in mean ICP between patients with poor and good outcome (12.8 ± 5.9 vs 11.8 ± 5.3 [mm Hg], p=0.3). Patients with poor outcomes were older than those with good outcomes (54 ± 19 vs 42 ± 16 [years], p<0.01).

Both PSI and AmpICP individually demonstrated a significant, though moderate, association with poor outcome six months after TBI (PSI: χ²(1)=5.69, p<0.02, AUC=0.61; AmpICP: χ²(1)=10.45, p<0.01, AUC=0.64). When accounting for age, PSI became redundant to the logistic regression model. Multivariate model incorporating both AmpICP and age had a good association with poor outcome (χ²(2)=22.25, p<0.01, AUC=0.70).

PSI gradually increased with rising ICP (Supplementary Fig. 4.2.a) and advanced age (Supplementary Fig. 4.2.b) for both patients with poor and good outcomes, with the increase in PSI being more pronounced with age than with mean ICP. AmpICP showed an upward trend with rising mean ICP in both patient groups (Supplementary Fig. 4.2.c). However, in contrast to PSI, there was no clear relationship between age and AmpICP in either group, regardless of outcome (Supplementary Fig. 4.2.d).

*Short discussion*

The findings regarding the relationship between poor outcomes and morphological parameters are similar to those observed for mortality. The only notable difference is the absence of distinctly elevated PSI values at low ICP levels in patients with poor outcomes, which were clearly evident in those who died. This difference may be explained by the younger age of patients with poor outcomes compared to those who died (54 ± 19 vs. 63 ± 15 [years]).


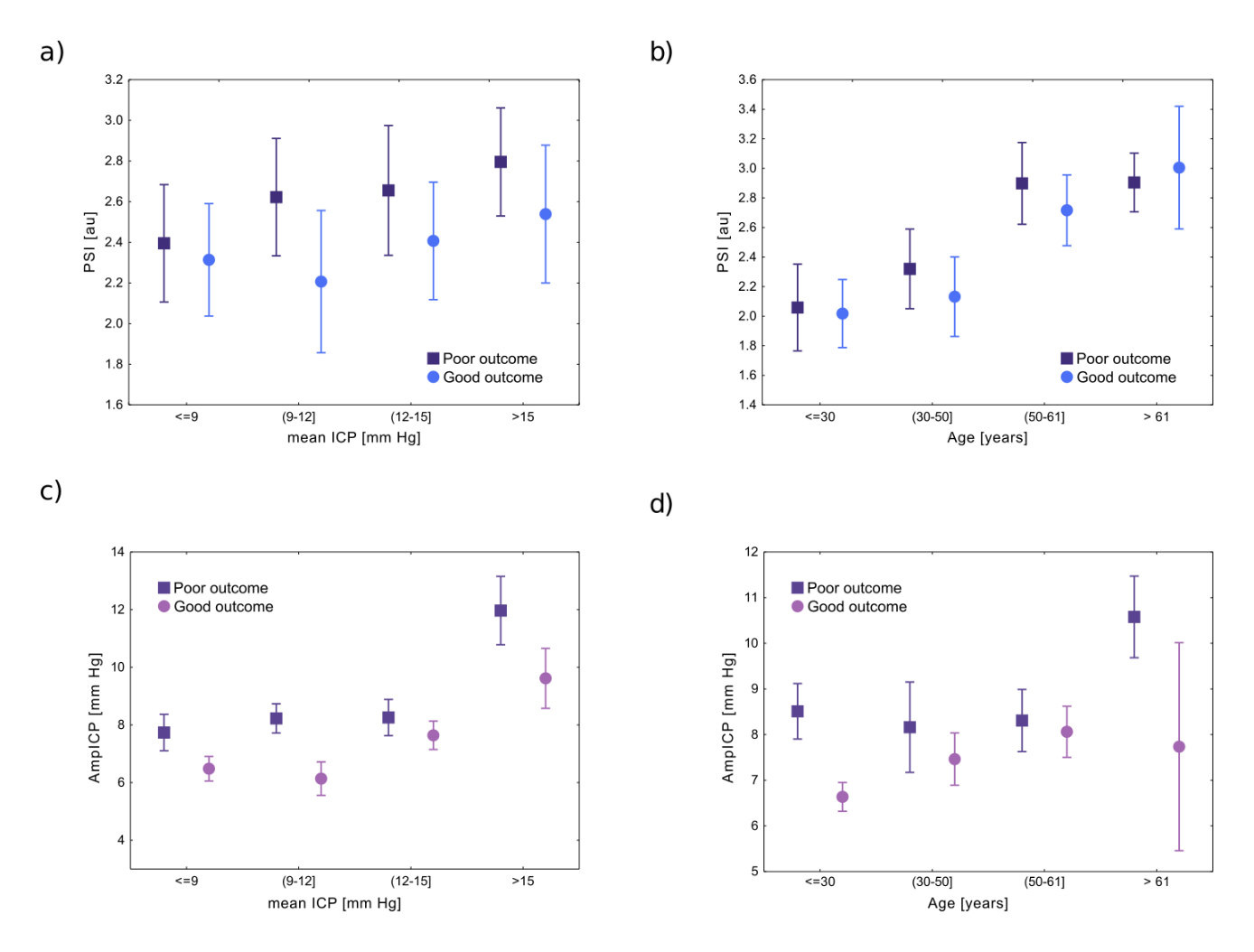


Supplementary Fig. 4.2. Differences in pulse shape index (PSI; upper panel) and peak-to-peak amplitude of intracranial pressure (ICP) pulse waveform (AmpICP; lower panel) accounting for mean ICP (subplots a and c) and age (subplots b and d) between patients with poor and good outcome six months post-injury. The central points in the graph represent the means, and the vertical bars denote the standard error. au—arbitrary units
