# Supplementary material for: Impact of age and mean intracranial pressure on the morphology of intracranial pressure waveform and its association with mortality in traumatic brain injury
Source: Crit Care. 2025 Feb 17;29:78. doi: 10.1186/s13054-025-05295-w (PMC11834513; doi:10.1186/s13054-025-05295-w)
Supplement: Supplementary file 5 — Additional file5 (DOCX 17 KB) [file 13054_2025_5295_MOESM5_ESM.docx]

**Supplementary material 5**

*Impact of systemic arterial blood pressure and its amplitude on ICP pulse morphology*

Arterial blood pressure (ABP) was measured via radial or femoral arterial lines connected to pressure transducers (Baxter Healthcare Corp. CardioVascular Group, Irvine, Ca, USA). Mean ABP was calculated for each patient as the average value of ABP recording lasting up to seven days post-injury. Amplitude of ABP pulse waveform (AmpABP) was calculated as the difference between the highest (maximum peak) and the lowest (minimum valley) values of the ABP pulse waveform within nonoverlapping 2-second windows.

The main effects of mean ABP (categorized into four levels: ≤ 78 mm Hg, (78–84] mm Hg, (84–91] mm Hg, and > 91 mm Hg) and AmpABP (categorized into four levels: ≤ 68 mm Hg, (68–75] mm Hg, (75–87] mm Hg, and > 87 mm Hg) as well as their interaction effects on AmpICP and PSI were investigated using factorial ANOVA. Correlation analysis was performed using the Pearson correlation coefficient (r).

The results showed that mean ABP had a significant effect on AmpICP (F(3, 167)=4.39, p<0.01), with an effect size of 0.073, indicating that mean ABP explained 7.3% of the variance in AmpICP. Similarly, AmpABP significantly influenced AmpICP (F(3, 167)=5.60, p<0.01), with an effect size of 0.091, i.e. explaining 9.1% of the variance in AmpICP. The interaction effect of AmpABP and mean ABP on AmpICP was not significant (F(9, 167)=1.52, p=0.14).

Regarding PSI, mean ABP had a significant effect (F(3, 167)=6.95, p<0.01), with an effect size of 0.111, indicating that 11.1% of the variance in PSI was explained by mean ABP. In contrast, the effect of AmpABP on PSI was not significant (F(3, 167)=0.55, p=0.65). The interaction effect of AmpABP and mean ABP on PSI was also not significant (F(9, 167)=1.05, p=0.06).

AmpABP showed a significant but weak correlation with age (r=0.22, p<0.01), while mean ABP was not associated with age. Both mean ABP and AmpABP were moderately correlated with AmpICP (r=0.32, p<0.01; r=0.35, p<0.01, respectively). PSI was moderately correlated with mean ABP (r=0.33, p<0.01) and weakly correlated with AmpABP (r=0.18, p=0.01).

*Short discussion*

Existing research indicates a moderate correlation between the amplitude of ICP pulse waveforms and ABP pulse waveforms [1–3]. In our study, consistent with previous findings, the amplitude of systemic ABP had a small impact on the amplitude of the ICP pulse waveform, but it did not affect its shape. On the other hand, mean ABP influenced both the shape and amplitude of the ICP pulse waveform. However, in this study we did not consider the influence of the ABP pulse waveform shape on ICP pulse morphology, focusing solely on its amplitude and mean value. An earlier study suggested a possible link between the shapes of ABP and ICP pulse waveforms [4], but this relationship remains to be addressed in further works.

*References*

1. Eide PK, Sorteberg W. Association among intracranial compliance, intracranial pulse pressure amplitude and intracranial pressure in patients with intracranial bleeds. Neurol Res. 2007;29:798–802. doi: 10.1179/016164107X224132

2. Evensen KB, Eide PK. Mechanisms behind altered pulsatile intracranial pressure in idiopathic normal pressure hydrocephalus: role of vascular pulsatility and systemic hemodynamic variables. Acta Neurochir (Wien). 2020;162:1803–13. doi: 10.1007/s00701-020-04423-5

3. Howells T, Lewén A, Sköld MK, Ronne-Engström E, Enblad P. An evaluation of three measures of intracranial compliance in traumatic brain injury patients. Intensive Care Med. 2012;38:1061–8. doi: 10.1007/s00134-012-2571-7

4. Vrabie O, Faltermeier R, Schmidt NO, Brawanski A, Lang EW. Fully convolutional networks for pathophysiological intracranial pressure waveform classification. https://www.biorxiv.org/content/10.1101/2020.11.17.381517v3. 2021.
