# Supplementary material for: Impact of age and mean intracranial pressure on the morphology of intracranial pressure waveform and its association with mortality in traumatic brain injury
Source: Crit Care. 2025 Feb 17;29:78. doi: 10.1186/s13054-025-05295-w (PMC11834513; doi:10.1186/s13054-025-05295-w)
Supplement: Supplementary file 6 — Additional file6 (SVG 17 KB) [file 13054_2025_5295_MOESM6_ESM.docx]

**Supplementary material 6**

*Analysis of sensitivity and specificity*

Supplementary Tables 6.1 and 6.2 illustrate the classification performance of the models for mortality and poor outcome prediction, respectively.

*Mortality analysis*

The dataset for mortality analysis was imbalanced, with 30 patients who died (18.2%) and 135 patients who survived (81.8%). With the default threshold of 0.5, this imbalance led to models achieving high specificity but very low sensitivity, as shown in Supplementary Table 6.1.

Supplementary Table 6.1. Classification results for mortality

| Metric | Sensitivity | Specificity |
| --- | --- | --- |
| AmpICP | 16.7% | 98.5% |
| PSI | 3.3% | 100.0% |
| mICP | 6.7% | 99.6% |
| Age | 16.7% | 97.8% |
| Age + AmpICP | 36.7% | 97.0% |

These results indicate that the models correctly identified the majority of survivors but failed to detect many cases of mortality. This is a common challenge when working with highly imbalanced datasets, where models tend to favour the majority class.

*Poor outcome analysis*

The dataset for poor outcome analysis was balanced, with 88 patients experiencing poor outcomes (53.3%) and 77 having good outcomes (46.7%). All of the models used a default threshold of 0.5. This resulted in a trade-off between sensitivity and specificity, as seen in Supplementary Table 6.2.

Supplementary Table 6.2. Classification results for poor outcomes

| Metric | Sensitivity | Specificity |
| --- | --- | --- |
| AmpICP | 63.6% | 58.4% |
| PSI | 68.2% | 37.7% |
| mICP | 82.9% | 22.1% |
| Age | 70.4% | 49.3% |
| Age + AmpICP | 64.7% | 58.4% |

The sensitivity values for poor outcomes were considerably higher than those for mortality, demonstrating the impact of a more balanced dataset on the model's performance. However, the trade-off between sensitivity and specificity reflects the inherent challenges of classification in clinical data and can be better tracked when looking at the ROC curves, which provide both sensitivity and specificity scores for different thresholds. While we agree that the sensitivity and specificity of the final model would be important when deploying it to be used in the clinical setting, for our comparison purposes AUC provides more information, combining the sensitivity and specificity for different thresholds.
